# Effect of Benzydamine Hydrochloride on Dental Plaque Accumulation During Short-Term Restricted Oral Hygiene: A Randomized, Placebo-Controlled Trial

**DOI:** 10.3390/ph19071013

**Published:** 2026-06-30

**Authors:** Ivan Puhar, Tina Paleško Tubikanec, Sabina Glavina, Matija Borovac, Domagoj Vražić, Ana Badovinac, Larisa Musić

**Affiliations:** 1 Department of Periodontology, University of Zagreb School of Dental Medicine, Gundulićeva 5, 10000 Zagreb, Croatia; 2 Poliklinika Šlaj-Anić, Štoosova Ulica 26, 10000 Zagreb, Croatia; 3 Elysium Dental d.o.o., Ulica Ivana Rendića 27, 10000 Zagreb, Croatia

**Keywords:** benzydamine, dental plaque, oral hygiene, anti-Inflammatory agents, randomized controlled trial

## Abstract

**Background/Objectives:** Benzydamine hydrochloride (B-HCl) is a non-steroidal anti-inflammatory agent with antimicrobial properties that may be beneficial in oral biofilm control. The aim of this study was to evaluate the effect of a 0.15% B-HCl mouthrinse on dental plaque accumulation and gingival inflammation under short-term conditions of restricted mechanical oral hygiene. **Methods:** Fifty periodontally healthy female subjects (aged 16–27 years) were randomly assigned (1:1) to receive either a 0.15% B-HCl mouthrinse or a placebo. Following professional prophylaxis, subjects rinsed with 15 mL twice daily for 30 s and refrained from all other oral hygiene procedures for 3 days. Full-Mouth Plaque Score (FMPS) was the primary outcome, and Full-Mouth Bleeding Score (FMBS) was the secondary outcome, recorded at baseline (Day 0) and Day 3. This study was conducted in accordance with CONSORT guidelines. **Results:** All subjects (n = 50) completed the study. FMPS increased significantly in both groups (*p* < 0.001). However, plaque accumulation at Day 3 was significantly lower in the B-HCl group compared with placebo (47.9% vs. 73.8%, *p* < 0.001), representing an absolute reduction of 25.9% and a relative reduction of 35.1%. No statistically significant differences were observed between groups in FMBS at Day 3 (*p* = 0.180). **Conclusions:** A 0.15% B-HCl mouthrinse reduced dental plaque accumulation compared with placebo during a 3-day period of restricted mechanical oral hygiene (mean difference: 25.9%; 95% CI: 16.1% to 35.7%). Given the short study duration, the anti-inflammatory properties of B-HCl could not be adequately evaluated. Longer-term studies are needed to determine whether B-HCl provides clinically meaningful benefits as an adjunct to mechanical oral hygiene. Trial registration: ClinicalTrials.gov (Identifier: NCT07565766).

## 1. Introduction

Dental plaque is a complex microbial community that forms on the tooth surfaces shortly after cleaning, as the enamel becomes coated with a conditioning layer known as the acquired pellicle [1]. This pellicle, composed primarily of proteins and glycoproteins, determines bacterial adhesion and subsequent plaque development [2,3]. The initial attachment of bacteria to the pellicle is reversible and mediated by weak physicochemical forces, allowing microorganisms to adhere to the tooth surface. This close contact enables stronger, more durable adhesion through specific molecular interactions between bacterial surface structures and complementary receptors in the pellicle [4,5]. As bacteria proliferate and interact, a spatially and functionally organized biofilm develops, characterized by increased thickness, structural complexity, and resistance to mechanical disruption [6,7]. An important component of dental plaque is the extracellular matrix, produced by both bacterial and host sources, including saliva and gingival exudate, which retains bacterial products and contributes to biofilm stability.

The accumulation and maturation of dental plaque trigger changes within the gingival tissues, affecting the vasculature, connective tissue, and junctional epithelium [8]. Clinically, this manifests as gingival inflammation (gingivitis) characterized by edema, erythema, bleeding, and tenderness [8,9]. Plaque-induced gingivitis is a reversible condition that responds well to effective plaque control. However, if plaque accumulates, changes in the subgingival biofilm may promote dysbiosis, increasing the risk of progression to periodontitis [8,10]. This close relationship between plaque accumulation and gingival inflammation shows the fundamental role of oral hygiene in maintaining periodontal health [10,11].

Mechanical plaque control using a toothbrush and interdental cleaning devices is the basic approach to biofilm control, but their effectiveness is highly dependent on patient compliance, manual dexterity, and proper technique [12]. In clinical practice, these conditions are often not fully achieved [13], resulting in inadequate plaque removal, particularly in interdental areas and on lingual tooth surfaces. Consequently, residual plaque frequently persists, promoting the development of gingival inflammation [14]. Due to these limitations, chemical plaque-control agents are commonly used as adjuncts to mechanical oral hygiene procedures. Their main role is preventative, as they inhibit bacterial adhesion and proliferation, thereby reducing the risk of gingivitis [15,16]. The effectiveness of chemical agents is determined by their antimicrobial spectrum and substantivity, defined as the ability to bind to oral surfaces and remain active over time. Among the numerous agents evaluated for chemical plaque control, chlorhexidine (CHX) is considered the reference standard due to its broad antimicrobial spectrum and high substantivity [17,18]. Nevertheless, its long-term use is associated with adverse effects such as tooth staining, taste disturbances, and desquamations of the oral mucosa [18,19]. Consequently, research has focused on developing alternative chemical agents with comparable potency but with fewer adverse effects. A wide range of substances has been investigated, including quaternary ammonium compounds, phenolic compounds and essential oils, bisbiguanide derivatives, oxygen-releasing agents, metal salts, enzymes, detergents, amine alcohols, fluorides, natural products, and other antiseptic agents [20]. Despite demonstrated antimicrobial or anti-inflammatory activity, many of these substances are limited by reduced substantivity, a narrow antimicrobial spectrum, or local side effects. Benzydamine hydrochloride (B-HCl) is a non-steroidal anti-inflammatory drug with local anesthetic activity [21]. Its mechanism of action is based on inhibiting the synthesis and activity of inflammatory mediators, stabilizing the membranes of platelets and other inflammatory cells, and reducing the release of arachidonic acid from membrane phospholipids [22]. Beyond its anti-inflammatory effects, B-HCl also exhibits direct antimicrobial activity against oral bacteria and inhibits biofilm formation in vitro, providing a biological rationale for evaluating its potential as a plaque-control agent [21,23].

Despite its use in various oral mucosal conditions, the role of B-HCl in preventing plaque-induced gingival inflammation remains unclear. Therefore, the aim of this randomized controlled trial was to evaluate the effect of a 0.15% B-HCl mouthrinse, compared with placebo, on dental plaque accumulation and gingival inflammation over a 3-day period of restricted mechanical oral hygiene. The hypothesis was that B-HCl would reduce plaque accumulation compared with placebo, yet the anti-inflammatory effect would not be observed due to the short-term period of mechanical hygiene disruption.

## 2. Results

Fifty subjects were enrolled in this study, 25 per study arm. All subjects were female, aged 16–27 years. All subjects successfully completed the study, and no adverse effects, including taste disturbance, burning sensation, or mucosal irritation, were reported or observed in either group.

Table 1 presents the inter-group differences in FMPS and FMBS at baseline (Day 0) and at the final (Day 3) measurement.

At baseline, FMPS was balanced between the two groups. At the time of final measurements, FMPS was significantly lower in the B-HCl group than in the placebo group (47.9% vs. 73.8%, *p* < 0.001).

FMBS was statistically higher at baseline in the B-HCl group (10.8%) compared to the control group (8.1%; *p* = 0.027). At the time of final measurements, there were no statistically significant differences in FMBSs between the two groups. To account for the baseline imbalance in FMBS (B-HCl: 10.8% vs. placebo: 8.1%), an ANCOVA was performed with Day-3 FMBS as the dependent variable, treatment group as the fixed factor, and baseline FMBS as the covariate. After adjustment for baseline FMBS, no significant treatment effect was observed (F(1,47) = 0.002, *p* = 0.962, partial η^2^ < 0.001). The adjusted marginal mean FMBS at Day 3 was 12.05% (95% CI: 9.56–14.54) in the B-HCl group and 12.15% (95% CI: 9.66–14.64) in the placebo group, yielding an adjusted mean difference of −0.10 percentage points (95% CI: −4.02 to 3.82). Baseline FMBS was a significant covariate (F(1,47) = 13.07, *p* = 0.001, partial η^2^ = 0.218), confirming the appropriateness of the adjustment. The ANCOVA results are presented in Table S3.

For the primary outcome (FMPS at Day 3), the mean between-group difference was 25.9 percentage points (95% CI: 16.1% to 35.7%), corresponding to a relative reduction of 35.1% compared with placebo. Cohen’s d was 1.54 (95% CI: 0.89 to 2.19). For the secondary outcome (FMBS at Day 3), the mean between-group difference was 3.4 percentage points (95% CI: −1.9% to 8.7%), with a Cohen’s d of 0.39 (95% CI: −0.17 to 0.94).

Intra-group changes between baseline (Day 0) and final (Day 3) measurements are shown in Table 2.

FMPS increased significantly in both groups. This increase was smaller in the B-HCl group (28.7%) than in the placebo group (59.5%). There were no significant changes in FMBS in either group.

Exploratory subgroup analyses based on smoking status were conducted due to small subgroup sizes (B-HCl group: 12 smokers, 13 non-smokers; placebo group: 7 smokers, 18 non-smokers). The complete subgroup data are presented in Supplementary Tables S1 and S2.

## 3. Discussion

This study investigated the effect of 0.15% B-HCl mouthrinse on dental plaque accumulation and gingival inflammation in young, periodontally healthy females. After three days of mechanical oral hygiene cessation, both groups showed increased plaque accumulation. However, plaque scores were significantly lower in the B-HCl group than in the placebo group (47.9% vs. 73.8%, *p* < 0.001), representing a relative reduction of approximately 35%. Plaque accumulation in the B-HCl group nevertheless increased substantially from baseline (19.2% to 47.9%), indicating that the mouthrinse reduced, but did not prevent, plaque formation under conditions of complete mechanical oral hygiene cessation. No statistically significant effect on gingival bleeding was observed. Interpretation of this outcome is limited by the short study duration. According to the classical experimental gingivitis model described by Page and Schroeder [24], the initial inflammatory lesion typically requires 4–10 days to develop. Consequently, the 3-day duration of the present study was likely insufficient to allow development of clinically detectable inflammatory changes. Therefore, the absence of a significant difference in FMBS should not be interpreted as evidence against the anti-inflammatory properties of B-HCl.

Due to its antimicrobial properties, the effects of B-HCl were evaluated in multiple studies with various designs and patient populations. Herrera et al. conducted a clinical trial in a population similar to ours: periodontally healthy younger adults [25]. The study evaluated a mouthrinse containing both B-HCl and cetylpyridinium chloride (CPC) and reported the greatest plaque growth inhibition in the group receiving the combined formulation. However, because two active agents were used simultaneously, the specific contribution of B-HCl cannot be determined. Therefore, direct comparisons with the present study, which evaluated only B-HCl, should be interpreted cautiously. Nevertheless, both studies support the concept that chemical agents can partially reduce plaque accumulation during short-term periods of restricted mechanical oral hygiene. In our study, B-HCl produced a modest plaque-reducing effect but did not prevent plaque formation, indicating that it may serve only as an adjunct to, rather than a replacement for, mechanical plaque control.

In another randomized study by Bozkurt et al., periodontally healthy subjects were using oral sprays containing CHX, B-HCl, or their combination, during a one-week non-brushing period [26]. The study reported that B-HCl alone was less effective compared with CHX or the CHX/B-HCl combination, and that adding B-HCl to CHX did not provide additional clinical benefits. All groups showed a significant increase in plaque and gingival parameters during the restricted oral hygiene period, indicating that neither individual nor combined antiseptic agents can fully prevent plaque accumulation in the absence of mechanical plaque control. Importantly, the combination CHX/B-HCl was associated with a higher incidence of burning sensations.

The effects of B-HCl can further be beneficial when combined with mechanical plaque control. Seshan et al. [27] evaluated the effect of 0.15% B-HCl mouthrinse in patients with chronic generalized gingivitis, comparing it to 0.2% CHX as an adjunct to routine oral hygiene. The results of both groups demonstrated statistically significant reductions in plaque index, gingival index, and bleeding scores after one month, with B-HCl showing efficacy comparable to CHX.

The antimicrobial properties of B-HCl have been confirmed in several studies. An early in vitro study by Fanaki et al. [21] demonstrated the antimicrobial activity of B-HCl against a broad spectrum of microorganisms, including Gram-positive and Gram-negative bacteria, yeasts, and fungi. Pina Vaz et al. [28] and Ardizzoni et al. [23] confirmed its antifungal activity against C. albicans, an important early colonizer involved in plaque maturation. This mechanism is consistent with the reduced plaque accumulation (47.9% vs. 73.8%) observed in our B-HCl group, though we did not measure C. albicans colonization. Ardizzoni et al. also showed that B-HCl reduces biofilm formation in vitro. Our in vivo findings, specifically the 25.9% absolute reduction in FMPS, are consistent with this anti-biofilm effect, although direct comparison is limited by differences between in vitro and clinical conditions. Further support comes from Wesley et al. [29], who demonstrated microbiological and clinical improvements following B-HCl irrigation in patients with chronic periodontitis, including reductions in motile bacterial species and sustained decreases in gingival inflammation and bleeding.

Exploratory analyses of smoking subgroups should be interpreted with caution due to the limited sample sizes, particularly the placebo smoker subgroup (n = 7). Although previous studies have suggested that smoking may suppress clinical signs of inflammation [30,31,32,33,34] no firm conclusions regarding smoking-related differences can be drawn from the present data. Any observed patterns should be considered hypothesis-generating only and require confirmation in adequately powered prospective studies.

This study has several limitations that should be acknowledged. The period of restricted oral hygiene was limited to three days. Although this duration was sufficient to evaluate plaque accumulation, it was likely too short to allow development of clinically detectable gingival inflammation, thereby limiting assessment of the anti-inflammatory properties of B-HCl. Ethical approval restricted the duration of oral hygiene cessation to three days, and longer experimental gingivitis models were therefore not feasible. An additional limitation relates to sample size estimation. Because no previous trial directly comparing B-HCl alone with a placebo was available, the calculation was based on the closest available reference study evaluating B-HCl in combination with CPC. Although this approach was reasonable for planning purposes, the expected effect size may not fully reflect the effect of B-HCl alone. In addition, the final sample size was slightly lower than the estimated target due to practical recruitment constraints. Therefore, the study should be considered exploratory. Limited statistical power, particularly for secondary outcomes such as FMBS, cannot be excluded. Another limitation of the present study is the baseline imbalance in FMBS (B-HCl: 10.8% vs. placebo: 8.1%, *p* = 0.027), which was significantly higher in the B-HCl group before treatment initiation. Although the absolute difference was modest and the change in FMBS over the 3-day period was comparable between groups, ANCOVA analysis was performed. The findings indicate that B-HCl did not significantly affect gingival bleeding during the 3-day experimental period. The study was conducted at a single center and included exclusively young, periodontally healthy female participants. While this homogeneous sample reduced biological variability and was appropriate for an exploratory study, it limits the generalizability of the findings to males, older adults, patients with periodontal disease, and broader populations. Additional limitations include the absence of an active comparator, in particular chlorhexidine, which prevents direct comparison with the current gold standard for chemical plaque control, and the lack of formal assessment of blinding effectiveness, which may have been influenced by the characteristic taste and mild anesthetic properties of B-HCl. Finally, no microbiological analyses were performed; therefore, the mechanisms underlying the observed reduction in plaque accumulation remain speculative. The statistical analyses were based on pre-specified non-parametric methods because the study variables did not meet assumptions of normality. Although mixed-effects or repeated-measures models may also be used in repeated-measures randomized studies, particularly for direct assessment of time-by-treatment interactions, the present approach was considered appropriate for the exploratory design, sample size, and distribution of the data. Future studies with larger samples and longer follow-up periods may benefit from the application of longitudinal modelling techniques.

Overall, the results of this study indicate that a 0.15% B-HCl mouthrinse can reduce dental plaque accumulation during short-term periods of restricted mechanical oral hygiene. However, the limited duration of the study did not allow a full assessment of its anti-inflammatory effects. Future studies with longer follow-up periods and more heterogeneous patient populations are needed to better define the long-term efficacy and broader applications of B-HCl in oral health management.

## 4. Materials and Methods

### 4.1. Patient Population

Subjects were screened from female subjects attending the Women’s General Gymnasium in Zagreb and the School of Dental Medicine, University of Zagreb. Young females were selected for practical recruitment reasons. All eligible subjects were screened with an oral examination and enrolled if they met the inclusion and exclusion criteria. Inclusion criteria were: (1) dentate subjects with at least 24 healthy teeth, and (2) no history of periodontal disease, confirmed by a clinically healthy periodontium, defined as probing pocket depth (PPD) ≤ 3 mm and no interdental clinical attachment loss. Exclusion criteria were: (1) pregnant or lactating females, (2) using hormonal contraceptives, (3) reporting systemic diseases or pharmacological treatment that could affect gingival inflammation, or (4) use of antibiotics within 6 months before the study. Subjects were eligible for enrolment irrespective of their smoking status. All subjects were informed about the study and provided written consent. Parental or legal guardian consent was obtained for underage subjects. The study was conducted in accordance with the ethical principles of the World Medical Association Declaration of Helsinki and was approved by the Ethics Committee of the School of Dental Medicine, University of Zagreb (05-PA-26-22/13). The trial was retrospectively registered at ClinicalTrials.gov (Identifier: NCT07565766; registration date: 23 April 2026).

### 4.2. Study Design

This study was a single-center, randomized, double-blind, placebo-controlled exploratory trial designed to evaluate the effect of a 0.15% benzydamine hydrochloride (B-HCl) mouthrinse Tantum Verde (Sanochemia Pharmazeutika AG, Neufeld/L, Austria) on plaque accumulation and gingival bleeding during a 3-day period of restricted mechanical oral hygiene. The study was conducted between March and April 2013.

The primary outcome was the Full-Mouth Plaque Score (FMPS), and the secondary outcome was the Full-Mouth Bleeding Score (FMBS).

Subjects were randomly assigned in a 1:1 ratio to receive either the active B-HCl rinse or a placebo rinse. The B-HCl and the placebo mouthrinse were similar in packaging, color, viscosity and in taste. The randomization sequence was computer-generated by an investigator not involved in clinical measurements or data analysis. Allocation codes were concealed in sequentially numbered, opaque, sealed envelopes and opened only by a designated study investigator responsible for dispensing the assigned rinse. Both subjects and the examiner were blinded to group allocation throughout the study period.

Oral hygiene was evaluated using the Plaque Control Record (O’Leary, 1972 [35]) at six sites on the gingival margin, determined after staining application of a plaque-disclosing solution (GC, Tri Plaque ID Gel, Tokyo, Japan). Full Mouth Plaque Score (FMPS) was expressed as the proportion of sites positive for plaque [(number of positive sites/number of all measured sites) × 100]. Gingival inflammation was assessed using bleeding on probing (BOP) (Ainamo & Bay, 1975 [36]) and dichotomously recorded at six sites per tooth. Full Mouth Bleeding Score (FMBS) was consequently calculated as described for FMPS. Both subjects and the clinical examiner were masked to group allocation to minimize potential bias and ensure the integrity of the study results.

All clinical measurements were performed by a single calibrated examiner. Examiner calibration was established before study initiation using repeated periodontal measurements on two separate occasions, with agreement within 2 mm in at least 98% of sites. FMPS and FMBS were subsequently recorded using a standardized examination protocol. No changes to the study design, outcomes, or analysis methods were made after trial commencement.

### 4.3. Study Protocol

**Figure 1:**
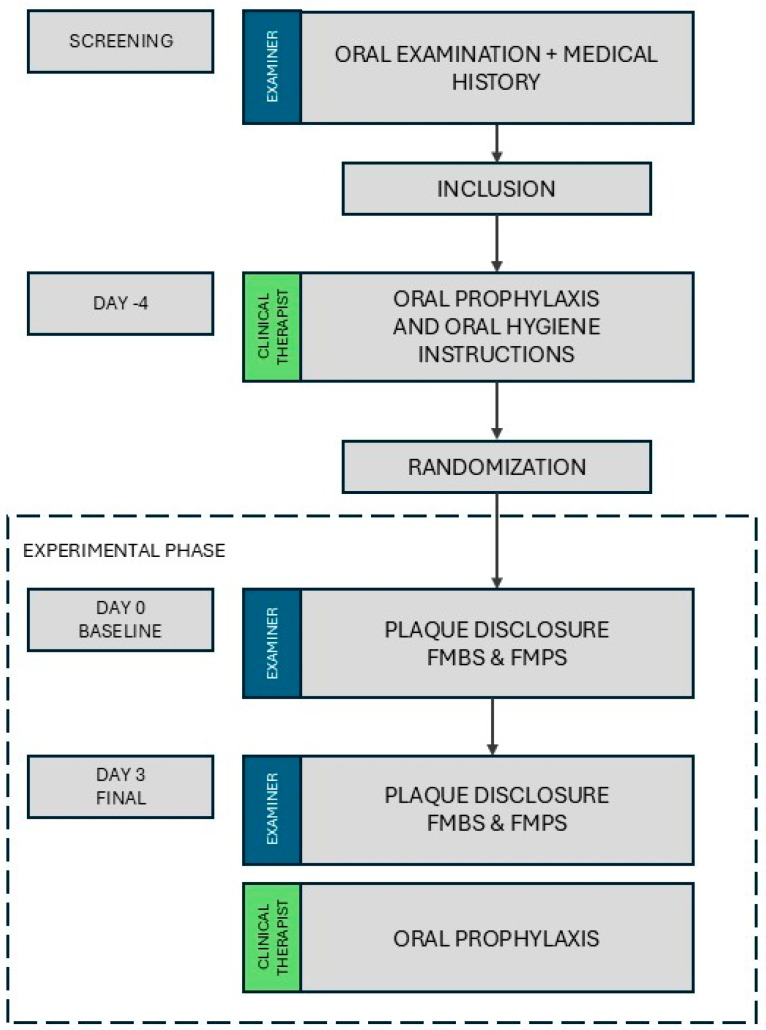
Study protocol and timeline (FMPS, Full-Mouth Plaque Score; FMBS, Full-Mouth Bleeding Score).

The study protocol is presented in Figure 1.

All enrolled subjects were seen four days before the study intervention (Day −4). A clinical therapist provided oral hygiene instructions and training. Oral prophylaxis was performed using an airscaler device KaVo SONICflex (Kavo Dental GmbH, Biberach, Germany), and professional polishing with a rotary brush and prophylactic paste. The period from Day −4 to Day 0 (the start of the study) was used to ensure a balanced clinical presentation of subjects before the study intervention.

The study intervention was performed on Day 0. FMPS and FMBS were measured and recorded. Subjects then received the assigned mouthrinse, with instructions to rinse 15 mL twice daily for 30 s, according to the manufacturer’s instructions. During the three-day follow-up, subjects were asked to refrain from all other oral hygiene procedures. Adverse events were monitored throughout the study period, and subjects were instructed to maintain a journal, reporting any unusual sensations or discomfort. At the final visit (Day 3), FMPS and FMBS were re-evaluated, marking the end of the experimental phase. Oral prophylaxis was then performed to re-establish a clean oral environment.

### 4.4. Sample Size Calculation

The sample size was estimated based on the study by Herrera et al. [25], which reported a mean difference in plaque index of 0.42 (SD 0.562) between B-HCl combined with CPC and placebo. As no prior direct comparison of B-HCl alone versus placebo was available, this represented the closest available estimate. Using these parameters with a two-sided alpha of 0.05 and 80% power, a sample size of 28 per group would be required. Recognizing the exploratory nature of this study and practical recruitment constraints, 25 subjects per group were enrolled.

### 4.5. Data Analysis

The qualitative variables in this study were group assignment (experimental or control), measurement point (first or second), and smoking status (yes or no); these were considered as independent variables in all analyses. The continuous dependent variables were the FMPS and FMBS. Descriptive statistics for the dependent variables were calculated for all combinations of independent variables.

For all analyses, the null hypotheses (defined by the study objectives) assumed no differences in the dependent variables, while alternative hypotheses indicated the presence of differences. Because the continuous variables did not meet the assumption of normality (Kolmogorov–Smirnov test), non-parametric tests were used. The Mann–Whitney U test was used to compare two independent samples (e.g., experimental and control groups), and the Wilcoxon signed-rank test was used for paired samples (e.g., first and second measurements). The significance level for rejecting the null hypothesis was set at α = 0.05. Results are presented in tables and figures. Statistical analyses were performed using IBM SPSS Statistics Software (version 18). No missing data occurred during the study. All randomized subjects completed the study and were included in the final analysis.

## 5. Conclusions

Within the limitations of the study, a 0.15% B-HCl mouthrinse significantly reduced dental plaque accumulation compared with placebo (absolute reduction: 25.9%; relative reduction: 35.1%). However, substantial plaque remained (47.9% of surfaces), and no significant effect on gingival bleeding was observed. Given the study design, no conclusions on anti-inflammatory properties can be drawn. Longer-term studies in more diverse populations are needed to determine whether B-HCl provides clinically meaningful benefits as an adjunct to mechanical oral hygiene.

## Figures and Tables

**Table 1:** Inter-group comparisons of FMPS and FMBS between the B-HCl and placebo groups at baseline (Day 0) and final (Day 3) measurements (Mann–Whitney U-test).

Variable	Measurement	Sample	n	Mean	Standard Deviation	Mann–Whitney U-Test		
						Mean Ranks	U	*p*
FMPS	Baseline	B-HCl	25	19.2	9.64	29.3	218.5	0.068
		Placebo	25	14.3	6.37	21.7		
	Final	B-HCl	25	47.9	16.51	16.3	82.5	<0.001
		Placebo	25	73.8	17.16	34.7		
FMBS	Baseline	B-HCl	25	10.8	5.48	30.0	199.0	0.027
		Placebo	25	8.1	5.48	21.0		
	Final	B-HCl	25	13.8	7.65	28.3	243.5	0.180
		Placebo	25	10.4	5.05	22.7		

**Table 2:** Intra-group changes in FMPS and FMBS from baseline (Day 0) to final (Day 3) measurements in the B-HCl and placebo groups (Wilcoxon signed-rank test).

Variable	Sample	Measurement	n	Mean	Standard Deviation	Wilcoxon Test		
						Mean Ranks	Z	*p*
FMPS	B-HCl	Baseline	25	19.2	9.64	4.0	−4.26	<0.001
		Final	25	47.9	16.51	13.4		
	Placebo	Baseline	25	14.3	6.37	0.0	−4.37	<0.001
		Final	25	73.8	17.16	13.0		
FMBS	B-HCl	Baseline	25	10.8	5.48	10.9	−1.48	0.141
		Final	25	13.8	7.65	13.4		
	Placebo	Baseline	25	8.1	5.48	9.3	−1.87	0.061
		Final	25	10.4	5.05	15.5		

## Data Availability

The data presented in this study are available from the corresponding author upon reasonable request, subject to ethical and privacy restrictions.

## Supplementary Materials

The following supporting information can be downloaded at: https://www.mdpi.com/article/10.3390/ph19071013/s1. Table S1: Comparisons of FMPS and FMBS according to smoking status within the B-HCl and placebo groups at baseline (Day 0) and final (Day 3) measurements (Mann-Whitney U-test); Table S2: Intra-group changes in FMPS and FMBS from baseline (Day 0) to final (Day 3) measurements according to smoking status in the B-HCl and placebo groups (Wilcoxon signed-rank test); Table S3: Analysis of covariance (ANCOVA) for Full-Mouth Bleeding Score (FMBS) at Day 3, with baseline FMBS as covariate and treatment group as fixed factor.

## Abbreviations

The following abbreviations are used in this manuscript:

B-HCl	Benzydamine hydrochloride
FMPS	Full-Mouth Plaque Score
FMBS	Full-Mouth Bleeding Score
CPC	Cetylpiridinium chloride
CHX	Chlorhexidine
