# Supplementary material for: Effect of Benzydamine Hydrochloride on Dental Plaque Accumulation During Short-Term Restricted Oral Hygiene: A Randomized, Placebo-Controlled Trial
Source: Pharmaceuticals (Basel). 2026 Jun 30;19(7):1013. doi: 10.3390/ph19071013 (PMC13414605; doi:10.3390/ph19071013)
Supplement: Supplementary file 1 [file pharmaceuticals-19-01013-s001.zip › pharmaceuticals-4329320-supplementary.pdf]

Supplementary Materials

Table S1 presents changes in FMPS and FMBS from baseline to final measurements by smoking status.

Table S1. Comparisons of FMPS and FMBS according to smoking status within the B-HCl and placebo groups at baseline (Day 0) and final (Day 3) measurements (Mann-Whitney U-test).

Variable	Sample	Measurement	Smoking	n	Mean	Standard deviation	Mann-Whitney U-test		
							Mean ranks	U	*P*
FMPS	B-HCl	Baseline	Yes	12	21.5	9.36	14.6	58.5	*0.295*
			No	**13**	17.2	9.79	11.5		
		Final	Yes	12	47.3	20.33	12.4	70.5	*0.689*
			No	13	48.5	12.88	13.6		
	Placebo	Baseline	Yes	7	16.8	7.49	15.4	46.5	*0.326*
			No	18	13.3	5.83	12.1		
		Final	Yes	7	81.6	18.90	16.4	39.0	*0.158*
			No	18	70.8	15.96	11.7		
FMBS	B-HCl	Baseline	Yes	12	13.2	6.36	16.5	36.5	***0.022***
			No	13	8.7	3.54	9.8		
		Final	Yes	12	14.5	6.71	14.5	60.5	*0.347*
			No	13	13.1	8.65	11.7		
	Placebo	Baseline	Yes	7	11.7	7.28	17.9	29.0	***0.041***
			No	18	6.6	4.00	11.1		
		Final	Yes	7	13.3	4.16	17.6	30.5	***0.047***
			No	18	9.3	5.04	11.2		

There were no statistically significant differences in FMPS between baseline and final measurements in either group (smokers or non-smokers).

For FMBS, in the B-HCl group, smokers presented with significantly higher baseline scores. This difference was not observed at the final measurement. In the placebo group, smokers showed significantly higher bleeding scores at baseline and at final measurement.

Intra-group differences between smokers and non-smokers in the experimental and placebo groups are shown in Table 4. FMPS values

increased significantly in both groups from baseline to final measurements, irrespective of the smoking status. The changes in FMBS values were not statistically significant in either subgroup. Analyses regarding smoking status were exploratory and were not pre-specified in the study protocol.

Table S2. Intra-group changes in FMPS and FMBS from baseline (Day 0) to final (Day 3) measurements according to smoking status in the B-HCl and placebo groups (Wilcoxon signed-rank test).

Variable	Sample	Smoking	Measurement	n	Mean	Standard deviation	Wilcoxon test		
							Mean ranks	Z	*P*
FMPS	B-HCl	Yes	Baseline	12	21.5	9.36	2.0	-2.90	***0.004***
			Final	12	47.3	20.33	6.9		
		No	Baseline	13	17.2	9.79	0.0	-3.18	***0.001***
			Final	13	48.5	12.88	7.0		
	Placebo	Yes	Baseline	7	16.8	7.49	0.0	-2.37	***0.018***
			Final	7	81.6	18.90	4.0		
		No	Baseline	18	13.3	5.83	0.0	-3.73	***<0.001***
			Final	18	70.8	15.96	9.5		
FMBS	B-HCl	Yes	Baseline	12	13.2	6.36	5.4	-0.51	*0.610*
			Final	12	14.5	6.71	7.6		
		No	Baseline	13	8.7	3.54	6.0	-1.65	*0.099*
			Final	13	13.1	8.65	6.7		
	Placebo	Yes	Baseline	7	11.7	7.28	4.8	-0.76	*0.446*
			Final	7	13.3	4.16	3.7		
		No	Baseline	18	6.6	4.00	5.5	-1.81	*0.070*
			Final	18	9.3	5.04	12.7		

Table S3. Analysis of covariance (ANCOVA) for Full-Mouth Bleeding Score (FMBS) at Day 3, with baseline FMBS as covariate and treatment group as fixed factor.

	**Sum of Squares**	**df**	**Mean Square**	**F**	**p-value**	**Partial η²**	**Observed Power**

Baseline FMBS (covariate)	533.8	1	533.8	13.07	**0.001**	0.218	0.943
Treatment group	0.1	1	0.1	0.002	**0.962**	<0.001	0.050
Error	1919.4	47	40.84				
Total	2453.3	49					

Abbreviations: df, degrees of freedom; FMBS, Full-Mouth Bleeding Score; B-HCl, benzydamine hydrochloride. Baseline FMBS was a significant covariate ($p = 0.001$). The treatment group effect was not significant ($p = 0.962$). Adjusted means are estimated marginal means controlling for baseline FMBS. Observed power was computed using $\alpha = 0.05$.
